# The role of serum testosterone and dehydroepiandrosterone sulfate in kidney function and clinical outcomes in chronic kidney disease: a systematic review and meta-analysis

**DOI:** 10.1530/EC-22-0061

**Published:** 2022-05-12

**Authors:** Anna C van der Burgh, Samer R Khan, Sebastian J C M M Neggers, Ewout J Hoorn, Layal Chaker

**Affiliations:** 1 Department of Internal Medicine, Erasmus University Medical Center, Rotterdam, the Netherlands; 2 Department of Epidemiology, Erasmus University Medical Center, Rotterdam, the Netherlands

**Keywords:** testosterone, DHEAS, kidney function, estimated glomerular filtration rate, chronic kidney disease, mortality, systematic review, meta-analysis

## Abstract

**Objective/design:**

Testosterone might mediate sex differences in kidney function and chronic kidney disease (CKD). However, few studies analyzing the association between testosterone and kidney function showed conflicting results. Therefore, we performed a systematic review and meta-analysis.

**Methods:**

Six electronic databases were searched from inception to March 4, 2020, for studies that investigated the association of (i) testosterone status with kidney function in the general population or (ii) testosterone status with clinical outcomes (kidney function decline, kidney failure, cardiovascular (CV) events, and cardiovascular and all-cause mortality) in CKD patients. We used random and fixed-effect models to obtain pooled effect estimates with 95% confidence intervals (CIs).

**Results:**

No randomized–controlled trials that met the inclusion criteria were identified. One study was conducted in the general population and reported an increased risk of incident CKD with low vs normal testosterone (hazard ratio (HR): 1.38, 95% CI: 1.05;1.80). Seven studies were conducted in men with CKD and included testosterone as determinant, of which six could be meta-analyzed. Low testosterone was associated with an increased risk of all-cause mortality and CV events (pooled HR: 1.98, 95% CI: 1.36;2.89; pooled HR of 2.40, 95% CI: 1.22;4.71, respectively). Two studies showed an increased risk of all-cause mortality with decreased dehydroepiandrosterone sulfate (DHEAS) in men with CKD; results regarding CV events were conflicting.

**Conclusions:**

Although literature is scarce, evidence suggests that lower testosterone may increase CKD risk in the general population and risk of all-cause mortality and CV events in men with CKD. Whether testosterone supplementation could prevent these potential detrimental outcomes should be determined in future intervention studies.

## Introduction

Chronic kidney disease (CKD) is a highly prevalent condition (1) and is often characterized by a decline in kidney function over time. Generally, a higher prevalence of CKD is found in women, while men with CKD have a higher rate of kidney function decline. This phenomenon is often referred to as the ‘CKD paradox’ (2, 3), and differences in sex hormones between men and women might underlie these existing sex differences (3, 4, 5).

In men, the major sex hormone is testosterone. However, literature regarding the effects of testosterone on the kidney has shown conflicting results. On the one hand, it is suggested that testosterone has harmful effects on the kidney, such as glomerular and tubular damage, kidney fibrosis, proteinuria, and hypertensive effects (6, 7, 8, 9, 10, 11). However, these effects were only found in animal studies in which testosterone depletion with or without consecutive testosterone replacement was investigated. On the other hand, testosterone is suggested to positively affect the kidney, including vasodilatation in the renal vascular bed, decreased inflammation, and decreased kidney injury, which has been reported in both animal and human studies (12, 13, 14, 15, 16, 17, 18). Moreover, dehydroepiandrosterone sulfate (DHEAS), a product of the testosterone precursor hormone DHEA, has also been suggested to affect the kidney through several mechanisms, but it is unclear if the net effect on kidney function decline is positive or negative (19, 20, 21, 22).

Prior studies assessing the association between serum testosterone and kidney function in the general population as well as the association between serum testosterone and clinical outcomes in a CKD population are few and have heterogeneous results. In addition, these studies have never been systematically analyzed, which could result in increased power to investigate the associations of interest. However, knowledge about the association between serum testosterone and kidney function could provide more insight into the mechanism behind the existing sex differences in kidney function decline and could reveal a potential therapeutic target to prevent this decline (i.e. testosterone supplementation in men with mild hypogonadism at high risk of CKD). We therefore set out to investigate the association of serum testosterone – as well as its precursors and active metabolites, if available – with kidney function decline and CKD. Additionally, we investigated the association of testosterone, its precursors, its active metabolites, and its supplementation with clinical outcomes in patients with CKD.

## Methods

### Study search and eligibility criteria

This systematic review and meta-analysis was conducted in accordance with the Preferred Reporting Items for Systematic Reviews and Meta-Analyses (PRISMA) guidelines for transparent reporting (Supplementary Material 1, see section on supplementary materials given at the end of this article). A systematic search within PubMed, Medline (OvidSP), Web-of-Science, Cochrane, PubMed publisher, and Google Scholar from inception to March 4, 2020, was conducted by a trained medical librarian from the Erasmus MC using a comprehensive search strategy (Supplementary Material). No language or date restrictions were applied. In addition, reference lists of potentially eligible studies and reviews concerning our topic were screened to identify additional eligible studies. We aimed to identify prospective cohort studies, case–control studies, or randomized–controlled trials (RCTs) that satisfy one of two predefined sets of eligibility criteria. First, to determine the association of testosterone status with kidney function (decline) or incident CKD in the general population, we searched for studies that satisfy the following criteria: (i) assessment of testosterone status at baseline in subjects aged above 18 years and (ii) prospective assessment of kidney function decline or moderate or severe CKD (KDIGO 2012 stage G3–G5 (23)) incidence as an outcome in patients with normal or low-normal kidney function (KDIGO 2012 stage G1–2) or in the general population. We included studies that defined testosterone status as serum levels of testosterone or hyper/hypogonadism, as well as levels of precursors and active metabolites of testosterone, if available. Studies involving subjects with moderate or severe CKD only were excluded. Secondly, to determine the association of testosterone status or testosterone replacement therapy with clinical outcomes in patients with moderate or severe CKD, we searched for studies that satisfy the following criteria: (i) assessment of testosterone status at baseline in patients with moderate or severe CKD, aged above 18 years and (ii) at least one prospective assessment of clinical outcomes of CKD. We included studies that reported on the following clinical outcomes: kidney function decline, kidney failure, cardiovascular (CV) events, CV mortality, and all-cause mortality.

### Study selection, data extraction, and quality assessment

Two reviewers (ACB and SRK) independently reviewed the titles and abstracts of the retrieved studies for suitability based on the selection criteria. Full texts of the potential eligible studies were retrieved and independently reviewed by the same two reviewers according to the eligibility criteria. If full texts were not available, we contacted the corresponding authors. Disagreements within the reviewing process were resolved by reaching consensus or by consulting a third reviewer (LC). A standardized, predefined data extraction form was used to extract relevant information from the included studies, including study design and setting, number and characteristics of subjects, testosterone status and assessment, kidney function assessment and CKD stage, clinical outcomes in subjects with CKD if applicable, and study quality. Corresponding authors were contacted in case of questions regarding the data analysis reported in the studies. Quality assessment of the identified studies was performed by two reviewers independently using the Newcastle–Ottawa Scale (NOS) (24) for cohort and case–control studies, with a score ranging between 0 and 9, or the Cochrane risk of bias tool (25) for RCTs, where the overall risk of bias was scored as low, moderate, or high risk of bias.

### Statistical analyses

Meta-analyses were performed when two or more studies assessed the association of (i) testosterone status with kidney function or incident CKD in subjects with normal or low-normal kidney function or within the general population, or (ii) testosterone status or testosterone replacement therapy with clinical outcomes in patients with moderate or severe CKD. In addition, similar cut-offs for the categorization of serum testosterone in the different studies are required for the meta-analysis.

The used term ‘serum testosterone’ is about serum total testosterone, serum free testosterone is indicated as such. Reported CV events and mortality were assessed together as CV events, as not all studies may distinguish between fatal and non-fatal CV events. We preferentially extracted and analyzed the most adjusted hazard ratios (HRs) and 95% confidence intervals (CIs), although the second to last adjusted HRs were used when the most adjusted models were additionally adjusted for serum creatinine only. Odds ratios (ORs) were extracted if HRs were not reported. However, we performed predefined sensitivity analyses by excluding studies not reporting HRs. Other predefined sensitivity analyses, if possible based on the available information, were: (i) excluding testosterone replacement therapy users at baseline and during follow-up; (ii) excluding anti-androgen users at baseline and during follow-up; (iii) excluding patients with diabetes mellitus type 2, prostate cancer, and breast cancer at baseline; and (iv) excluding studies with only dialysis patients. If data were sufficient (≥2 studies per subgroup), subgroup analyses were performed for age, sex, body mass index (BMI), diabetes mellitus, CKD stage, study quality, and testosterone assay. Both random-effects models according to DerSimonian and Laird and fixed-effect models were used to calculate the pooled estimates and 95% CIs, and results were displayed using forest plots. Random-effect models were reported as the main results. Statistical heterogeneity was assessed by using the I^2^ statistic where I^2^ values of ≤25% indicate low, 25–50% moderate, and ≥50% high heterogeneity. Publication bias was assessed visually by using funnel plots and statistically by using an Egger test. All statistical analyses were performed in R statistical software (meta package, R-project, R Foundation for Statistical Computing (2020), 3.6.3).

## Results

### Study selection

**Figure 1:**
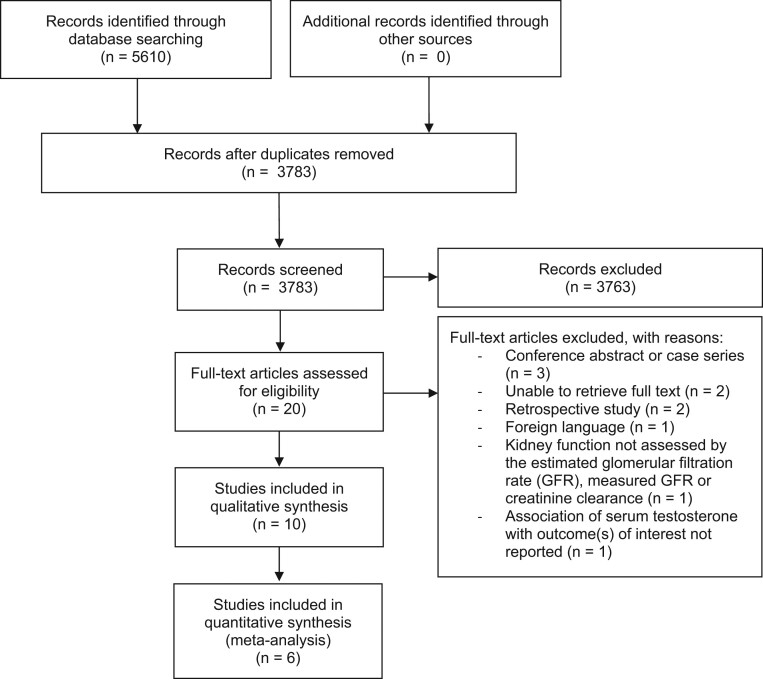
Flowchart of the study selection.

In total, 3783 reports were identified after removing duplicates, of which 3763 were excluded based on title and abstract (Fig. 1). After reading the full texts, ten additional reports were excluded, leaving ten reports that met the eligibility criteria and were included in the systematic review. Of these, six could be included in a meta-analysis.

### Study characteristics and quality assessment

We did not identify RCTs that met the inclusion criteria. All studies included testosterone or DHEAS as measure of testosterone status, and no studies including precursors or active metabolites of testosterone were identified. In addition, all studies were prospective cohort studies and were performed in Europe, Asia, Australia, and North America (Table 1). Out of the ten studies, only one investigated the association of testosterone status with kidney function (decline) or incident CKD in the general population (26). This study was published in 2019 and included 1277 participants. The other nine studies investigated the association of testosterone status with clinical outcomes in men with moderate or severe CKD (stage G3–G5), although one of them included men with mild CKD as well (stage G1–G2) (27). These studies were published between 2009 and 2015 and had sample sizes ranging between 94 and 623. None of the studies included kidney function decline or kidney failure as outcome. Eight out of ten studies included serum testosterone as a determinant, while the other two studies included serum DHEAS as a determinant. None of the studies investigated testosterone replacement therapy as a determinant. Half of the studies reported that none of the study participants used testosterone replacement or anti-androgen therapy, while the others did not provide any information on this. Testosterone cut-offs for hypogonadism ranged between 5.0 and 12.1 nmol/L, and follow-up duration varied from 20 months up to 11.2 years. Several methods for measuring serum testosterone were reported, including enzyme immunoassay (ELISA), liquid chromatography-tandem mass spectrometry, and radioimmunoassay (RIA) techniques. Serum DHEAS was measured by using chemiluminescent enzyme immunoassay or RIA techniques. All studies adjusted for a different set of covariates are displayed in Table 2. In general, the quality of studies was moderate to good with a score ranging from 6 to 9 (Supplementary Table 2).

**Table 1:** Characteristics of included studies on the association between testosterone status and kidney function, as well as testosterone status and mortality or CV events in CKD populations.

First author, journal, year of publication	Population	Year of study start	Age, mean ± SD or median (IQR)	Total, n*	Type of testosterone status measurement	Measurement method	Testosterone or DHEA-S, mean ± SD or median (IQR)
Amiri M, *Andrology*, 2019	Adult men from district-13 of Tehran, Iran	1999–2001	42 ± 13	1277	Testosterone	Enzyme-linked immunoassay	3.7 (3.0–5.2) nmol/L
Bello AK, *American Journal of Kidney Diseases*, 2014	Patients initiating HD therapy in one of three Canadian centers	2005	61 ± 15	623	Testosterone	Certified method unspecified	234.1 ± 146.1 ng/dL
Carrero JJ, *Journal of the American Society of Nephrology*, 2009	Patients receiving HD therapy at one of five dialysis units in Stockholm, Sweden	2003–2004	63 (49–73)	126	Testosterone	Certified method unspecified	286 (206–346) ng/dL
Carrero JJ, *Nephrology Dialysis Transplantation*, 2011	CKD stage 5, incident HD, and prevalent HD patients from Sweden	1999 and 2003	59 (48–67)	260	Testosterone	Certified method unspecified	11.0 (8.2–14.0) nmol/L
Grossman M, *Clinical Endocrinology*, 2015	Patients with CKD not on dialysis, patients on dialysis, and kidney transplant recipients from a single center in Melbourne, Australia	2003–2004	58 (50–72)	143	Testosterone	Validated liquid chromatography–tandem mass spectrometry	10.8 (8.1–14.4) nmol/L
Gungor O, *Clinical Journal of the American Society of Nephrology*, 2010	Patients on 3 weekly conventional HD from ten dialysis centers in Turkey	2005	54 ± 13	420	Testosterone	Enzyme-linked immunoassay	8.69 ± 4.10 nmol/L
Hsu HJ, *Experimental Gerontology*, 2012	Patients on HD for more than 6 months from one center in Taiwan	2007	59 ± 15	94	DHEAS	Radioimmunoassay	1237.3 ± 1312.7 ng/mL
Kakiya R, *Nephrology Dialysis Transplantation*, 2012	Patients on HD treatment at one center in Japan	2004	61 ± 10	313	DHEAS	Chemiluminescent enzyme immunoassay	771 (436–1351) ng/mL
Kyriazis J, *Nephrology Dialysis Transplantation*, 2011	Patients on HD therapy from three centers in Greece	2005–2006	65 ± 12	111	Testosterone	Radioimmunoassay	8.1 (IQR N.R.) nmol/L
Yilmaz MI, *Clinical Journal of the American Society of Nephrology*, 2011	Patients suspected of manifest renal failure in one medical center in Ankara, Turkey	2006–2010	54 ± 12	239	Testosterone	Radioimmunoassay	N.R.
**First author, journal, year of publication**	**Hypogonadism definition**	**Hypogonadism, *n*** (%)		**Type of kidney function measurement/CKD definition**		**All-cause mortality, *n*** (%)	**Incident cardiovascular events and mortality, *n*** (%)
Amiri M, *Andrology*, 2019	T <350 ng/dL	605 (47.4)		eGFR based on serum creatinine CKD: eGFR creatinine <60 mL/min per 1.73 m2		N.A.	N.A.
Bello AK, *American Journal of Kidney Diseases*, 2014	T borderline: 231–346 ng/dL T low: < 231 ng/dL	343 (55.1)		N.R.		166 (26.6)	Fatal and non-fatal CV events: 98 (15.7)
Carrero JJ, *Journal of the American Society of Nephrology*, 2009	T low-normal: 288–403 ng/dL T low: < 288 ng/dL**	T low-normal: 39 (31.0) T low: 66 (52.4)		N.R.		65 (51.6)	Fatal CV events: 38 (30.2)
Carrero JJ, *Nephrology Dialysis Transplantation*, 2011	T low-normal: 10–14 nmol/L T low: <10 nmol/L	T low-normal: 88 (33.8) T low: 112 (43.1)		CKD stage 5 definition N.R.		76 (29.2)	N.A.
Grossman M, *Clinical Endocrinology*, 2015	T intermediate: 5.0–11.9 nmol/L T low: < 5.0 nmol/L	T intermediate: 70 (49.0) T low: 16 (11.2)		CKD stage 3–4 definition N.R. General CKD: eGFR creatinine <60 mL/min per 1.73 m^2^		52 (36.4)	20 (14.0)
Gungor O, *Clinical Journal of the American Society of Nephrology*, 2010	T low-normal: 10–14 nmol/L T low: <10 nmol/L	T low-normal: 101 (24.0) Low T: 277 (66.0)		N.R.		104 (24.8)	N.A.
Hsu HJ, *Experimental Gerontology*, 2012	DHEAS < 790 ng/mL	N.R.		N.R.		35 (37.2)	Fatal CV events: 16 (17.0)
Kakiya R, *Nephrology Dialysis Transplantation*, 2012	N.R.	N.R.		N.R.		68 (21.7)	Fatal and non-fatal CV events: 118 (37.7)
Kyriazis J, *Nephrology Dialysis Transplantation*, 2011	T < 8 nmol/L	Low T: 54 (48.6)		N.R.		49 (44.1)	28 (25.2)
Yilmaz MI, *Clinical Journal of the American Society of Nephrology*, 2011	T ≤ 10 nmol/L	Low T: 78 (32.6)		CKD defined by K/DOQI guidelines, using eGFR creatinine and the presence of kidney injury (24 h proteinuria)		24 (10.0)	Fatal CV events: 22 (9.2) Non-fatal CV events: 50 (20.9)

^*Data in this column represents the total number of males in the included studies, as results were reported for males only. **^Definition using tertiles: T low-normal: 233–345 ng/dL; T low: < 233 ng/dL.

CKD, chronic kidney disease; CV, cardiovascular; DHEAS, DHEA sulfate; eGFR, estimated glomerular filtration rate; HD, hemodialysis; N.A., not applicable; N.R., not reported; SD, standard deviation; T, testosterone.

**Table 2:** Results of included studies on the association between testosterone status and kidney function, as well as testosterone status and mortality or CV events in CKD populations.

First author, journal, year of publication	Association testosterone with CKD, HR (95% CI)						Adjustments
	Continuous testosterone		Low vs normal testosterone		Quintiles of testosterone		
**First author, journal, year of publication**	Association testosterone with all-cause mortality, HR* (95% CI)			Association testosterone with CV events/mortality, HR* (95% CI)			**Adjustments**
General population, testosterone							
Amiri M, *Andrology*, 2019	0.82 (0.61;1.10)		1.38 (1.05;1.80)		Qu1 (lowest T); 1.58 (1.04;2.40) Qu2; 1.20 (0.77;1.85) Qu3; 1.14 (0.73;1.77) Qu4; 1.07 (0.70;1.66) Qu5 (highest T); Reference		Age, BMI, smoking, diabetes, dyslipidemia, hypertension
Continuous testosterone	Categorized testosterone	Tertiles of testosterone	Continuous testosterone	Categorized testosterone	Tertiles of testosterone
CKD population, testosterone							
Bello AK, *American Journal of Kidney Diseases*, 2014	0.58 (0.37;0.90) Per 10 ng/dL increase in log T	- T normal: reference - T borderline: 1.32 (0.72;2.42) - T low: 1.48 (0.82;2.66)	N.R.^**^	0.72 (0.36;1.42) Per 10 ng/dL increase in log T	- T normal: reference - T borderline: OR 1.61 (0.69;3.74) - T low: OR 1.38 (0.60;3.19)	N.R.^**^	Age, smoking status, BMI, SHBG, cancer, and diabetes
Carrero JJ, *Journal of the American Society of Nephrology*, 2009	N.R.	T< 33rd percentile (low) vs normal: 1.74 (1.01;3.02)	N.R.	N.R.	T< 33rd percentile (low) vs normal:2.47 (1.04;5.87)	N.R.	Age, SHBG, baseline CVD, diabetes, ACEI/ARB medication use, IL-6, and serum albumin
Carrero JJ, *Nephrology Dialysis Transplantation*, 2011	N.R.^#^	OR low vs normal T: 1.9 (1.0;3.9)	N.R.	N.R.	N.R.	N.R.	Age, diabetes, CRP, and kidney failure phase
Grossman M, *Clinical Endocrinology*, 2015	1) 0.93 (0.88;0.99) 2) 0.94 (0.88;0.99) Per 1 nmol/L increase	N.R.	N.R.	N.R.	N.R.	N.R.	(i) Age, renal disease status, BMI, and cardiac troponin T (ii) Age, diabetes, pre-existing CVD, renal disease status, BMI, CRP, and serum albumin
Gungor O, *Clinical Journal of the American Society of Nephrology*, 2010	0.96 (0.89;1.02) Per 1 nmol/L increase	N.R.	- Middle vs high: 0.76 (0.38;1.54) - Low vs high: 1.49 (0.83;2.66)	N.R.	N.R.	N.R.	Age, HD duration, diabetes, CVD, BMI, serum albumin, creatinine, and CRP
Kyriazis J, *Nephrology Dialysis Transplantation*, 2011	N.R.	Low vs normal: 2.81 (1.23;6.38)	Low vs middle+high: 4.04 (1.86;8.76)	N.R.	Low vs normal: 2.29 (0.78;6.72)	Low vs middle+high: 2.48 (0.90;6.85)	Age, BMI, baseline CVD, log HD vintage, serum albumin, log CRP, and pulse wave velocity
Yilmaz MI, *Clinical Journal of the American Society of Nephrology*, 2011	N.R.	N.R.	N.R.	0.83 (0.78;0.88) Per 1 nmol/L increase	N.R.	N.R.	Age, eGFR, diabetes, previous CVD, CRP, serum albumin, and flow-mediated dilation
**First author, journal, year of publication**	**Association DHEAS with all-cause mortality, HR** (95% CI)			**Association DHEAS with cardiovascular events/mortality, HR** (95% CI)			**Adjustments**
	Continuous DHEAS	Categorized DHEAS	Quartiles of DHEAS	Continuous DHEAS	Categorized DHEAS	Quartiles of DHEAS	
CKD population, DHEAS							
Hsu HJ, *Experimental Gerontology*, 2012	1.00 (1.00;1.00) Per 1 ng/mL increase	Low vs high: 3.84 (1.48–9.95)	N.R.	1.00 (1.00;1.00)	Low vs high: 3.99 (0.98;16.20)	N.R.	Age, baseline diabetes, chronic heart failure, COPD, cardiac thoracic ratio, hs-CRP, dialysis duration, and serum albumin
Kakiya R, *Nephrology Dialysis Transplantation*, 2012	N.R.	N.R.	Q1 vs Q2–4: 2.37 (1.37;4.08)	N.R.	N.R.	- Q1 vs Q3–4:1.96 (1.22;3.15) - Q2 vs Q3-4: 1.10 (0.68;1.77)	Age, dialysis vintage, diabetic nephropathy, BMI, serum albumin, log-CRP, pre-existing CVD, smoking, and hypertension

^*^Unless specified otherwise; ^**^Pattern of results of tertiles similar to categorized testosterone; ^#^Low T had impact on mortality risk, although weak.

ACEI, angiotensin-converting enzyme inhibitors; ARB, angiotensin receptor blockers; BMI, body mass index; CKD, chronic kidney disease; COPD, chronic obstructive pulmonary disease; (hs-) CRP, (high sensitivity) C-reactive protein; CV, cardiovascular; CVD, cardiovascular disease; eGFR, estimated glomerular filtration rate; HD, hemodialysis; HR, hazard ratio; IL-6, interleukin; N.R., not reported; Q, quartile; Qu, quintile; SHBG, sex-hormone-binding globulin; T, testosterone.

### Association of serum testosterone with incident chronic kidney disease

Only one study, including 1277 men, provided data on the association of serum testosterone with kidney function and included incident CKD as their outcome of interest (26). Increased levels of log-transformed serum testosterone were associated with a statistically non-significant decreased risk of incident CKD (HR: 0.82, 95% CI: 0.61;1.10, Table 2). In this study, serum testosterone was also categorized into low and normal testosterone using a cut-off of 350 ng/dL (12.1 nmol/L). Low testosterone was reported to be associated with an increased risk of incident CKD when compared to normal testosterone (HR: 1.38, 95% CI: 1.05;1.80, Table 2).

### Association of serum testosterone with all-cause mortality in subjects with CKD

Six studies reported on the association of serum testosterone with all-cause mortality in subjects with CKD (28, 29, 30, 31, 32, 33). Serum testosterone was investigated as a continuous parameter (*n*  = 3), a dichotomous categorical parameter (*n*  = 3), and a trichotomous categorical parameter (*n*  = 2).

### Continuous serum testosterone and all-cause mortality

**Figure 2:**
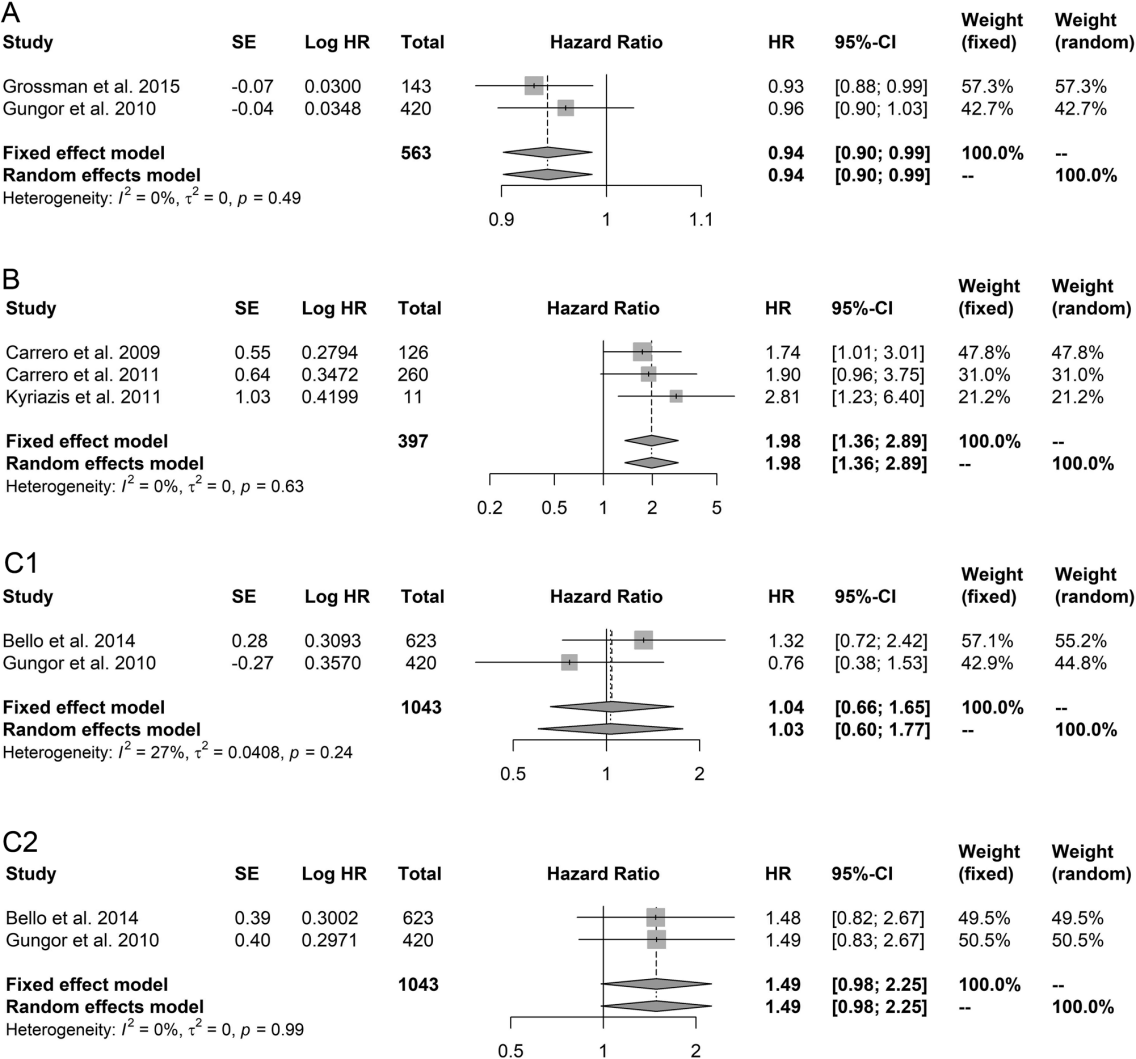
Forest plots of serum testosterone and all-cause mortality. (A) Association of continuous serum testosterone with all-cause mortality. (B) Association of two categories of serum testosterone with all-cause mortality: low vs reference. (C) Association of three categories of serum testosterone with all-cause mortality: (1). Borderline vs reference (2). Low vs reference. Hazard ratios with 95% CIs are delineated by squares with horizontal lines; pooled hazard ratios are delineated by diamonds.

In one (28) of the three studies that investigated the association between continuous serum testosterone and all-cause mortality, serum testosterone was log-transformed and increased levels of this log-transformed serum testosterone were associated with a decreased risk of all-cause mortality (HR: 0.58, 95% CI: 0.37;0.90, per 10 ng/dL, Table 2). Due to the log-transformation, this study could not be included in the meta-analysis. The corresponding author was contacted to request an investigation of the association using non-transformed serum testosterone, but responded that this was not possible due to the non-normal distribution of serum testosterone. The other two studies (31, 32) were included in the meta-analysis and higher serum testosterone levels were associated with a decreased risk of all-cause mortality when using a random-effects model, with a HR of 0.94 (95% CI: 0.90–0.99, Fig. 2A) and without evidence of heterogeneity (I^2^ of 0%, *P*-value = 0.49).

### Dichotomized serum testosterone and all-cause mortality

The three studies (29, 30, 33) that investigated the association between serum testosterone as a dichotomized parameter and all-cause mortality all used a different cut-off for low serum testosterone levels, i.e., 8.1 (30), 10.0 (29), and 8.0 nmol/L (33). Furthermore, the study using a cut-off of 10.0 nmol/L to define low serum testosterone was also the study that only reported ORs. All three studies individually reported that low serum testosterone was associated with an increased risk of all-cause mortality when compared to the reference, and an overall pooled HR of 1.98 (95% CI: 1.36–2.89, Fig. 2B) was shown, without evidence of heterogeneity (I^2^ of 0%, *P*-value = 0.63). Excluding the study reporting ORs only as a sensitivity analysis yielded similar results (overall pooled HR: 2.02, 95% CI: 1.28;3.18, Supplementary Fig. 1). One of the studies (33) also categorized serum testosterone using a cut-off of 5.2 nmol/L for low serum testosterone and reported that low serum testosterone was associated with a significantly increased risk of all-cause mortality when compared to normal serum testosterone (HR: 4.04, 95% CI: 1.86;8.76, Table 2). This study also showed that low serum free testosterone (cut-off: 0.21 nmol/L) was associated with a significantly increased risk of all-cause mortality when compared to normal serum free testosterone (HR: 2.62, 95% CI: 1.27;5.44).

### Trichotomized serum testosterone and all-cause mortality

Two studies categorized serum testosterone into three categories, i.e., low, borderline, and normal, although different cut-offs were used. One study (28) defined low and borderline serum testosterone as serum testosterone < 8.0 nmol/L and 8–12 nmol/L, respectively, while the other study defined low and borderline serum testosterone as serum testosterone < 10.0 nmol/L and 10–14 nmol/L, respectively (32). In the two studies, the reported HRs for the association between borderline vs normal serum testosterone and all-cause mortality were in the opposite direction, although significance was not reached in either study (HR: 1.32, 95% CI: 0.72;2.42 (28) and HR: 0.76, 95% CI: 0.38;1.54 (32), Table 2). After meta-analyzing the data using a random-effects model, a pooled HR of 1.03 (95% CI: 0.60;1.77) was shown, with moderate heterogeneity (I^2^ of 27%, *P*-value = 0.24, Fig. 2C1). The reported HRs for the association between low vs normal serum testosterone and all-cause mortality were similar, with a HR of 1.48 (95% CI: 0.82;2.66) (28) and a HR of 1.49 (95% CI: 0.83;2.66) (32) (Table 2). When comparing low vs normal serum testosterone, the pooled HR was 1.49 (95% CI: 0.98;2.25), without evidence of heterogeneity (I^2^ of 0%, *P*-value = 0.99), for all-cause mortality (Fig. 2C2).

### Association of serum testosterone with cardiovascular events in CKD patients

**Figure 3:**
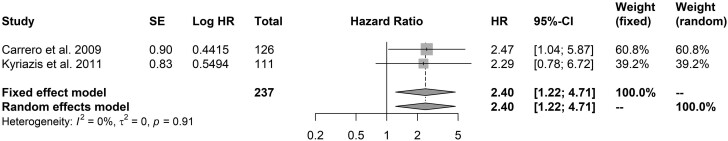
Forest plot of serum testosterone and CV events. Association of two categories of serum testosterone with CV events: low vs reference. Hazard ratios with 95% CIs are delineated by squares with horizontal lines; pooled hazard ratios are delineated by diamonds

Of the four studies that included data on the association of serum testosterone with CV events in subjects with CKD, two used continuous serum testosterone in their analyses (27, 28). Both showed that higher serum testosterone levels were associated with a decreased risk of CV events, both including non-fatal and fatal events. However, only one study provided an adjusted HR reaching statistical significance (HR: 0.83, 95% CI: 0.78;0.88, Table 2) (27). Furthermore, similar results were reported in this study on the association between serum free testosterone and CV events (HR: 0.65, 95% CI: 0.53–0.80). As one of the studies (28) log-transformed serum testosterone, findings could not be meta-analyzed. In the other two studies, serum testosterone was categorized into low and normal serum testosterone, with cut-offs of 8.1 (30) and 8.0 nmol/L (33). Low serum testosterone was associated with an increased risk of CV mortality when compared to normal serum testosterone, with a pooled HR of 2.40 (95% CI: 1.22;4.71) and without evidence of heterogeneity (I^2^ of 0%, *P*-value = 0.91, Fig. 3).

### Association of serum DHEAS with all-cause mortality and cardiovascular events

Two studies (34, 35) investigated the association of DHEAS with all-cause mortality and both showed that low DHEAS levels were associated with an increased risk of all-cause mortality; however, only when DHEAS was categorized (low vs high DHEAS, HR: 3.84, 95% CI: 1.48;9.95 (34), and quartile 1 (lowest) vs quartile 2–4(highest), HR: 2.37, 95% CI: 1.37;4.08 (35)). Due to large differences in categorization cut-offs, these findings could not be meta-analyzed. The same two studies also investigated the association of DHEAS with CV events; however, meta-analysis of this data was also not possible due to different expressions of the exposure, i.e., continuous vs categorical and difference in number of categories. One study (34) showed no significant association between DHEAS levels and the risk of CV mortality, neither when adding serum DHEAS as a continuous covariate to the model (HR: 1.00, 95% CI: 1.00;1.00) nor when adding serum DHEAS as a categorical covariate to the model (HR: 3.99, 95% CI: 0.98;16.20, low vs normal serum DHEAS) (Table 2). The second study (35) did report a significantly increased risk of CV events, including both non-fatal and fatal events, when comparing the first quartile of serum DHEAS to the reference consisting of the third and fourth quartile of serum DHEAS (HR: 1.96, 95% CI: 1.22;3.15), but not when comparing the second quartile of serum DHEAS to the same reference (HR: 1.10, 95% CI: 0.68;1.77) (Table 2).

### Evaluation of publication bias

Neither the visual assessment of the funnel plots (Supplementary Fig. 2) nor the Egger test (*P* > 0.2) showed signs of publication bias for the association between continuous and categorized serum testosterone and all-cause mortality. Similarly, no signs of publication bias for the association between categorized serum testosterone and CV events were shown (Supplementary Fig. 3, Egger test = 0.9145). Results should be interpreted with caution due to the limited number of included studies.

## Discussion

The current evidence on the association of serum testosterone with kidney function in the general population and with clinical outcomes in patients with CKD was compiled in this systematic review and meta-analysis. Only one study investigated the association between serum testosterone and kidney function in the general population and reported a 1.4-fold increased risk of CKD with lower serum testosterone levels (26). In addition, we report an almost 2-fold increased risk of all-cause mortality and a 2.4-fold increased risk of CV mortality with low serum testosterone levels in patients with CKD. Low serum DHEAS levels were associated with an increased risk of all-cause mortality, whereas conflicting results were shown for the association with CV events.

We identified one study that investigated the association of serum testosterone with incident CKD in the general population. In addition, we identified nine studies investigating the associations of serum testosterone (*n*  = 7) and serum DHEAS (*n*  = 2) with clinically relevant endpoints in CKD patients. However, these studies differed in many aspects, including study design, cut-off levels to define low serum testosterone, and definition of CV events. Furthermore, not all studies reported on the use of testosterone replacement therapy and all studies adjusted for a different set of covariates. Nevertheless, findings of our systematic review and meta-analysis suggest that higher serum testosterone is beneficial for the kidney. These results are supported by several animal and human studies investigating the effects of serum testosterone on the kidney (12, 13, 14, 15, 16, 17, 18), although detrimental effects of serum testosterone on the kidney have also been reported (6, 7, 8, 9, 10, 11). However, these studies were all conducted in animals in which testosterone depletion was effectuated. Several mechanisms explaining the potential beneficial effects of higher serum testosterone on the kidney have been proposed. Serum testosterone could exert positive effects on the kidney by causing vasodilatation in the renal vascular bed (12). Furthermore, in male rats that were exposed to renal ischemia, testosterone infusion attenuated the rise in plasma creatinine, urinary kidney injury molecule-1, and kidney inflammation, and prevented a reduction in outer medullary blood flow (13). More specifically, testosterone has been shown to reduce kidney inflammation by reducing the levels of inflammatory cytokines (13, 14). In addition, lower testosterone has been associated with lower hematocrit (15) and with an increased risk of anemia (16, 17), which in turn can play a role in kidney ischemia (18). The increased risk of all-cause mortality with lower testosterone could be explained by the reported increased risk of CV events in the current review. Moreover, low serum testosterone has been associated with CV risk factors and events such as diabetes (36, 37, 38), stroke (39, 40), metabolic syndrome (37, 41, 42), and atherosclerosis (43) in the general population. In patients with CKD, the same mechanisms could play a role as well. In addition, the question remains if serum testosterone is causally associated with mortality in this population or if the association could be explained by the underlying poor health status. That is, CKD itself can independently cause hypogonadism by disturbing the hypothalamic–pituitary–gonadal axis (44, 45) or by causing testicular damage such as interstitial fibrosis and calcification (45, 46). Therefore, hypogonadism might be considered as a surrogate marker of a greater degree of kidney dysfunction, which itself is associated with an increased risk of CV events and mortality, as well as with an increased risk of all-cause mortality.

As the association between serum testosterone and kidney function in the general population was investigated in only one study (26), results should be interpreted with caution and further studies are needed to replicate the results. This study reported an increased risk of CKD with lower serum total testosterone levels (26). However, the study was conducted in Iran, which could make the results less generalizable to other ethnic groups. Moreover, this study only included eGFR based on serum creatinine as an assessment of kidney function, which might not be the most optimal assessment of kidney function to investigate the association of interest because of the potential mediating role of muscle mass. That is, low serum testosterone is suggested to negatively affect muscle mass (47, 48, 49), and decreased muscle mass could decrease the levels of serum creatinine (50, 51) and therefore increase eGFR based on serum creatinine. As serum cystatin C is not affected by muscle mass, the use of serum cystatin C for calculating eGFR should ideally be considered when investigating the association between testosterone and kidney function. In contrast, a recent Mendelian randomization study that included a European population reported that higher levels of genetically predicted bioavailable serum testosterone in men were associated with an increased risk of both CKD and albuminuria and with lower eGFR based on creatinine or a combination of creatinine and cystatin C (52). An explanation for this discrepancy in findings might be found within the use of serum total testosterone vs serum bioavailable testosterone. This is supported by the fact that when total testosterone was included as the exposure in the Mendelian randomization study instead of bioavailable testosterone, the significance of all associations disappeared. Moreover, even though not statistically significant, higher levels of genetically predicted total testosterone seemed to be associated with a lower risk of CKD, which is comparable to the findings of the Iranian study.

Our results suggest that low serum testosterone is detrimental for kidney function. Our study is however not sufficient to address the causality of this association, and it is therefore too early to determine whether testosterone replacement therapy in men with low serum testosterone might have a protective effect on the kidney. Furthermore, testosterone replacement therapy also has potential adverse effects including an increased risk of CV events and fluid retention (53, 54, 55, 56, 57, 58). Studies investigating the effectiveness and safety of testosterone replacement therapy in patients with CKD or the association between testosterone replacement therapy and kidney function are limited (59, 60). Therefore, intervention studies are necessary to address causality and to assess the balance between efficacy and safety of testosterone replacement therapy.

The main strength of our study is that we performed an extensive literature search in multiple electronic databases with a limited amount of restrictions in order to provide an up-to-date and complete overview of the available literature on the association between serum testosterone and kidney function and on the association between serum testosterone and clinical outcomes in patients with CKD. However, our study also has some limitations. Despite the extensive literature search, the number of studies included in this systematic review and meta-analysis was low. This limited our possibilities to perform meta-analyses on all associations of interest and to perform stratification analyses. Furthermore, even though there were no signs of heterogeneity and publication bias in our study, we could not completely rule out their presence due to the low number of included studies. Different definitions of hypogonadism or adjustment for different confounders may have introduced heterogeneity. In addition, the included studies are all observational studies, so the presence of selection and information bias related to observational study designs as well as of confounding and reverse causality cannot be excluded. Finally, all included studies only included men or only provided results in men, which limits the generalizability of our findings. The generalizability of our findings is also limited by the age distribution of the participants in the included studies. The mean age of the participants in the studies investigating the association between testosterone status and clinical outcomes in CKD patients ranged between 54 and 65 years, and as testosterone levels decline with age, results might not be applicable to older populations.

This study shows that the evidence on the association between serum testosterone and incident CKD is sparse, with only one study describing an association between lower testosterone levels and an increased risk of CKD. Furthermore, investigation of the association of serum testosterone and clinical outcomes in patients with CKD revealed an increased risk of CV events and all-cause mortality with lower serum testosterone levels, although the number of studies was limited, study characteristics were heterogeneous, and both kidney function decline and kidney failure were not investigated as outcomes. Therefore, future studies are needed to investigate whether low serum testosterone is actually detrimental for the kidney and if testosterone replacement therapy could effectively and safely prevent these detrimental outcomes.

## Supplementary Material

Supplementary Material

## Declaration of interest

The authors declare that there is no conflict of interest that could be perceived as prejudicing the impartiality of the research reported.

## Funding

This work did not receive any specific grant from any funding agency in the public, commercial, or not-for-profit sector.
