# Supplementary material for: The role of serum testosterone and dehydroepiandrosterone sulfate in kidney function and clinical outcomes in chronic kidney disease: a systematic review and meta-analysis
Source: Endocr Connect. 2022 May 12;11(6):e220061. doi: 10.1530/EC-22-0061 (PMC9254301; doi:10.1530/EC-22-0061)
Supplement: Supplementary Material [file supplementary_material.pdf]

<u>Supplementary material</u>

Example of the electronic search strategy

Supplementary Table S1. PRISMA 2009 checklist.

Supplementary Table S2. Newcastle Ottawa Quality Assessment Scale for Cohorts of included studies

Supplementary Figure S1. Forest plots for categorized serum testosterone and all-cause mortality, excluding the study from Carrero et al. 2011

Supplementary Figure S2. Funnel plots for serum testosterone and all-cause mortality

Supplementary Figure S3. Funnel plot for serum testosterone and CV events

Example of the electronic search strategy

Medline (OvidSP)

(exp Testosterone/ OR Androsterone/ OR exp Dehydroepiandrosterone/ OR exp Hypogonadism/ OR (testosteron* OR androsteron* OR dehydroepiandrosteron* OR prasteron* OR androstenolon* OR hypogonadism* OR ((gonad*) ADJ3 (insufficienc* OR failure*))).ab,ti.) AND (Kidney Diseases/ OR Diabetic Nephropathies/ OR exp Nephritis/ OR Nephrosclerosis/ OR exp Renal Insufficiency, Chronic/ OR exp Kidney Function Tests/ OR (CKD OR GFR OR glomerulofiltration* OR ((glomerul* OR kidney OR renal*) ADJ3 (filtrat* OR hyperfiltrat* OR permeabilit*)) OR ((creatin*) ADJ3 (clearanc*)) OR glomerulpath* OR nephrosis* OR nephritis* OR nephropath* OR perinephritis* OR ((kidney OR perirenal* OR renal*) ADJ3 (disease* OR disorder* OR patholog* OR function* OR dysfunction* OR failure* OR ischemi*))).ab,ti.) NOT (exp animals/ NOT humans/)

Supplementary Table S1. PRISMA 2009 checklist.

Section/topic	#	*Checklist item*	*Reported on page #*
**Title**			
Title	1	Identify the report as a systematic review, meta-analysis, or both.	1
**Abstract**			
Structured summary	2	Provide a structured summary including, as applicable: background; objectives; data sources; study eligibility criteria, participants, and interventions; study appraisal and synthesis methods; results; limitations; conclusions and implications of key findings; systematic review registration number.	2
**Introduction**			
Rationale	3	Describe the rationale for the review in the context of what is already known.	3,4
Objectives	4	Provide an explicit statement of questions being addressed with reference to participants, interventions, comparisons, outcomes, and study design (PICOS).	3,4
**Methods**			
Protocol and registration	5	Indicate if a review protocol exists, if and where it can be accessed (e.g., Web address), and, if available, provide registration information including registration number.	5
Eligibility criteria	6	Specify study characteristics (e.g., PICOS, length of follow-up) and report characteristics (e.g., years considered, language, publication status) used as criteria for eligibility, giving rationale.	5
Information sources	7	Describe all information sources (e.g., databases with dates of coverage, contact with study authors to identify additional studies) in the search and date last searched.	5
Search	8	Present full electronic search strategy for at least one database, including any limits used, such that it could be repeated.	Supplementary material
Study selection	9	State the process for selecting studies (i.e., screening, eligibility, included in systematic review, and, if applicable, included in the meta-analysis).	5,6
Data collection process	10	Describe method of data extraction from reports (e.g., piloted forms, independently, in duplicate) and any processes for obtaining and confirming data from investigators.	6
Data items	11	List and define all variables for which data were sought (e.g., PICOS, funding sources) and any assumptions and simplifications made.	6
Risk of bias in individual studies	12	Describe methods used for assessing risk of bias of individual studies (including specification of whether this was done at the study or outcome level), and how this information is to be used in any data synthesis.	6
Summary measures	13	State the principal summary measures (e.g., risk ratio, difference in means).	6,7
Synthesis of results	14	Describe the methods of handling data and combining results of studies, if done, including measures of consistency (e.g., I^2) for each meta-analysis.	6,7
Risk of bias across studies	15	Specify any assessment of risk of bias that may affect the cumulative evidence (e.g., publication bias, selective reporting within studies).	6,7
Additional analyses	16	Describe methods of additional analyses (e.g., sensitivity or subgroup analyses, meta-regression), if done, indicating which were pre-specified.	6,7

Supplementary TableS1 (continued). PRISMA 2009 Checklist.

Section/topic	*#*	*Checklist item*	*Reported on page #*
**Results**			
Study selection	17	Give numbers of studies screened, assessed for eligibility, and included in the review, with reasons for exclusions at each stage, ideally with a flow diagram.	8, Figure 1
Study characteristics	18	For each study, present characteristics for which data were extracted (e.g., study size, PICOS, follow-up period) and provide the citations.	8, Table 1
Risk of bias within studies	19	Present data on risk of bias of each study and, if available, any outcome level assessment (see item 12).	8, Supplementary Table 2
Results of individual studies	20	For all outcomes considered (benefits or harms), present, for each study: (a) simple summary data for each intervention group (b) effect estimates and confidence intervals, ideally with a forest plot.	8-11, Table 2, Figure 2, Figure 3
Synthesis of results	21	Present results of each meta-analysis done, including confidence intervals and measures of consistency.	8-11, Figure 2, Figure 3
Risk of bias across studies	22	Present results of any assessment of risk of bias across studies (see Item 15).	12, Supplementary Figure 2, Supplementary Figure 3
Additional analysis	23	Give results of additional analyses, if done (e.g., sensitivity or subgroup analyses, meta-regression [see Item 16]).	Supplementary Figure 1
**Discussion**			
Summary of evidence	24	Summarize the main findings including the strength of evidence for each main outcome; consider their relevance to key groups (e.g., healthcare providers, users, and policy makers).	13-16
Limitations	25	Discuss limitations at study and outcome level (e.g., risk of bias), and at review-level (e.g., incomplete retrieval of identified research, reporting bias).	13-16
Conclusions	26	Provide a general interpretation of the results in the context of other evidence, and implications for future research.	13-16
**Funding**			
Funding	27	Describe sources of funding for the systematic review and other support (e.g., supply of data); role of funders for the systematic review.	17

Moher D, Liberati A, Tetzlaff J, Altman DG, The PRISMA Group (2009). Preferred Reporting Items for Systematic Reviews and Meta-Analyses: The PRISMA Statement. PLoS Med 6(6): e1000097. doi:10.1371/journal.pmed1000097

Supplementary Table S2. Newcastle Ottawa Quality Assessment Scale for Cohorts of included studies

First author, Journal, year of publication	Selection	Comparability	Outcome	Total
M. Amiri, Andrology, 2019	****	***	**	9/9
A.K. Bello, American Journal of Kidney Diseases, 2014	****	***	*	8/9
J. J. Carrero, Journal of the American Society of Nephrology, 2009	****	***	*	8/9
J. J. Carrero, Nephrology Dialysis Transplantation, 2011	****	***	*	8/9
M. Grossman, Clinical Endocrinology, 2015	****	**	*	7/9
O. Gungor, Clinical Journal of the American Society of Nephrology, 2010	****	***	-	7/9
H.J. Hsu, Experimental Gerontology, 2012	****	**	-	6/9
R. Kakiya, Nephrology Dialysis Transplantation, 2012	***	***	-	6/9
J. Kyriazis, Nephrology Dialysis Transplantation, 2011	****	**	*	7/9
M.I. Yilmaz, Clinical Journal of the American Society of Nephrology, 2011	***	**	*	6/9

Supplementary Figure S1. Forest plots for categorized serum testosterone and all-cause mortality, excluding the study from Carrero et al. 2011

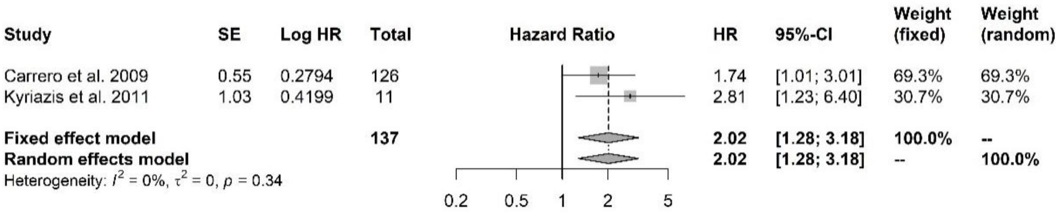

Hazard ratios with 95% confidence intervals are delineated by squares with horizontal lines; pooled hazard ratios are delineated by diamonds.

Supplementary Figure S2. Funnel plots for serum testosterone and all-cause mortality

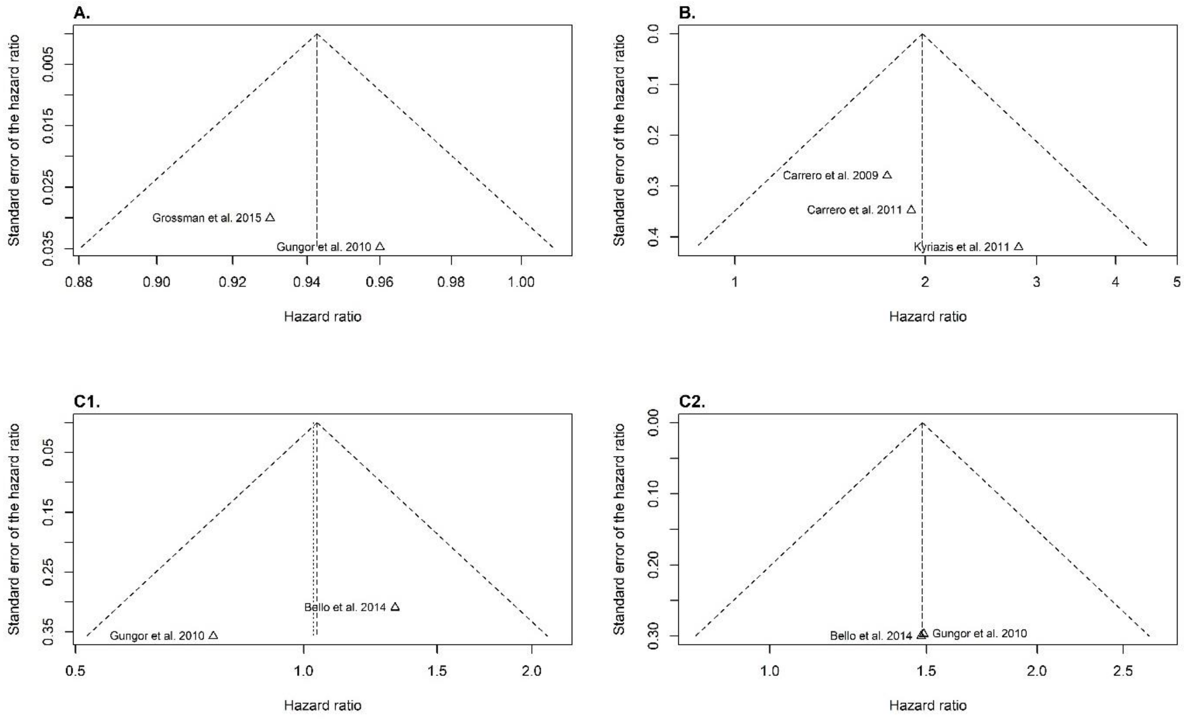

A. Association of continuous serum testosterone with all-cause mortality;P-value of the Egger test = 0.4897

B. Association of two categories of serum testosterone with all-cause mortality: low vs. reference;P-value of the Egger test = 0.6304

C. Association of three categories of serum testosterone with all-cause mortality:

1. Borderline vs. reference;P-value of the Egger test = 0.2425

2. Low vs. reference;P-value of the Egger test = 0.9873

Supplementary Figure S3. Funnel plot for serum testosterone and CV events

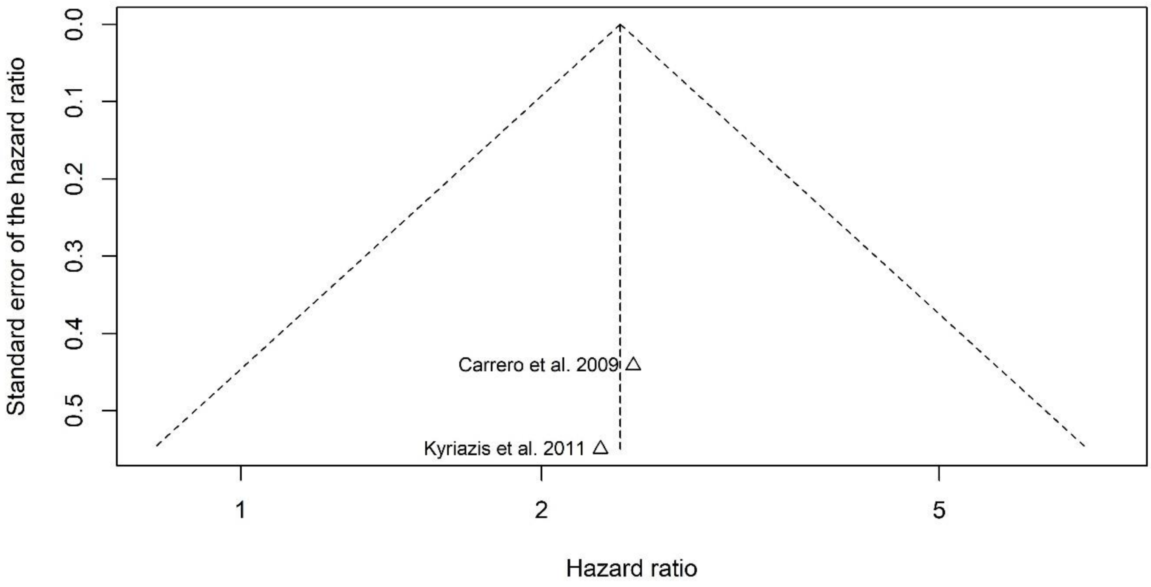

Association of two categories of serum testosterone with CV events: low vs. reference; P-value of the Egger test = 0.9145
